# The Line to Salonica

**Published:** 1920-04-17

**Authors:** 


### III.—WAR CONDITIONS IN THE NEAR EAST.

### The Line to Salonica.

It is a curious, though very instructive fact that human nature invariably seems to be compounded of the most perverse characteristics, displayed at the very times when they are the least appropriate. Thus in our small travelling compartment, where six of us were condemned to spend many hours in very close proximity, either your opposite neighbour malignantly stretched out his legs to their fullest extent, scraping your shins in the process, and with merely a grunt for an apology, as if he were the aggrieved rather than the aggriever. Or again, when after many attempts you at last succeeded in establishing a more or less comfortable position, and were just entering into that blissful region presided over by the sleepy monarch, suddenly you experienced a violent thump on your shoulder, and turning a sleepy look in that direction, found that your next companion had suddenly decided to use your person as a resting place. You protested in choice language, but without avail. The sleeper moved not, and probably to show his contempt at your feeble efforts, emitted most inharmonious snores, sinking at the same time more and more heavily against your shoulder, causing you immeasurable discomfort.

In such a manner we travelled, on one of the most tedious railway journeys that I have ever experienced, without relaxations other than eating and drinking, spinning yarns, and dreaming by fits and starts. Certainly on this journey speedy travelling was never even attempted, but to give the engine-drivers their due, they had to rely almost entirely on wood fuel.

### German Engineering.

We passed through ranges of mountains, whose grandeur was indeed impressive. I shall never forget the wonderful panorama presented by the sacred mountain of Olympus, surrounded by its attendant satellites; one could almost see Zeus himself seated on the summit dispensing justice to his world of worshippers. There were also a great number of long tunnels, in which, however, the air always seemed very fresh, but, our candles being only too scarce, the absence of gas and electric light was severely felt. This line had recently been built by German engineers, and they certainly deserve every credit for the admirable way in which they had overcome what must at first have appeared insuperable obstacles.

During the middle of the night one of my companions generously woke me up, and facetiously suggested that I should look out of the window. I did. Imagine a very dark night and the train now at a standstill on a viaduct, our carriage in the very centre of the latter, which bridged over a ravine whose depths seemed to be infinite. The bridge looked none too strong, and ours was a very long and heavy train, with an engine at both ends, as is the custom in these parts. Imagination is sometimes a horrible curse, and in this instance it certainly caused me to experience that cold shiver down the back which is the prerogative of the cowardly villain when he meets his doom in heavy drama. The Adjutant and myself went forward and interviewed the engine-driver, telling him that we considered that the train should be moved on to terra-firma. As, however, the rearmost engine was then taking in water, and seeing that both engines were themselves beyond the viaduct, this driver did not seem to realise any danger. One source of comfort was the fact that immediately in front of our coach there was a waggon full of French ammunition, the explosion of which would have speedily ended our tortures if anything untoward had happened.

Later, on we went again, and tried to be comfortable in sleep, but the limited space and the lack of soap and water were a considerable deterrent. The movements of this train were most eccentric; both engines made a great noise, and we were constantly being jolted on starting which, as we constantly stopped, was of frequent occurrence. It often seemed to me that the two engines antagonised rather than aided each others' efforts.

### Deep Ravines and Vast Plains.

There were, however, some compensations for the weary traveller, for much of the scenery was wonderful to behold, mountains towering over deep ravines, or vast plains stretching for many miles.

Now at last we saw some signs of man's habitations, and realised that we were approaching Salonica. Soon we left the city on our right, and, passing through French, Italian, and Greek camps, we proceeded to one of the British encampments, placed at some distance from the city. Far away in the distance were seen a range of mountains, which formed the northern bulwarks of this place, and hid from our eyes, though not from our imaginations, the land beyond, where so many brave fellows lie in their last sleep.

After a great amount of shunting we disembarked at Lembert Station. As usual, the darkness was rapidly coming on, but the welcome sight of some real, British railway engines, still painted with the well-known letters, took us for a moment into one of the great termini situated in the Euston Road, London!

Previous articles appeared on Feb. 7, p. 434, and March 6, p. 537.

